# Fluxion Dilutions

**Published:** 1881-06

**Authors:** Wm. Jefferson Guernsey

**Affiliations:** Phila.


### "FLUXION DILUTIONS."

WM. JEFFERSON GUERNSEY, M.D., PHILA.

AGAIN are we called upon to witness an attack upon the "*Fluxion dilutions.*" When a man openly pours out his little volley against the possibility of our reaching the C.M. or M.M. rounds of the ladder, we do not attempt to fight him. We object to "hitting a man when he's down," (very *low* down). But a double barrelled article, such as appeared in the *Hahnemannian Monthly*, for March, is too good to pass over lightly. Were it not for the kindly feeling which the writer holds toward his Maryland friend and old classmate he would be tempted to ask him if he ever *tried* a fluxion potency; or, in fact, any potency above a red, white or blue.

Our very good friend is not satisfied with flatly denying what he can not prove, that a very high potency does not exist, but savagely declares that they will not, or do not, *act*, that they are, in brief, "shams."

It is a strange "coincident" that since using these "shams" exclusively in our practice, and giving single, or very few doses of them, to a prescription, we have found our mortality list very much smaller in proportion to the cases treated than when we carried a larger pocket case, and loaded it up with the 3d and 4th dilutions. If we bring forward a case or two which has "ceased to be," so far as ills are concerned, and tell our friend that a high potency, and a fluxion potency *will* act, we trust that he will forbear any ill feeling toward the writer.

MISS L. G., æt. 19 years, complained of the following: soreness of throat, worse on swallowing; tough mucus collecting in throat; scraping rawness, and, worst of all, a sensation as though she had a "grater" in her throat. This latter symptom well described the appearance which was that of a marked case of follicular pharyngitis. The fauces were dark red, and she complained of more or less headache. All of these symptoms she had been suffering from for over four weeks, and they were becoming worse so rapidly as to alarm her. I gave her *one dose* of *argentun nit.* 45 M. (Fincke). Handed her Lac. Sac., Q S. to last a week, asking her to call in that time. She did so, stating that she only called because I had so requested; that her throat was better the second day after her first visit, and that it was now entirely well, which an inspection confirmed.

"Mr. M. G., æt. 22 years, told the following story of himself. He had felt well until two days ago, when he was "taken" with chilliness which was more or less severe for several hours, and had continued to some extent, up to within a short time of my visit; he was then in a state of intense heat. Severe aching of lumbar region. He said he felt as though his back would *break*." Intense sensation of fulness in forehead, skin very hot, pulse 120, full and bounding. Getting rapidly worse. Gave him one dose of *bell.* C.M. (F. C.) dry on the tongue, and left another to be taken in three hours. S. L. in water *ad libitum.* Next day he had no fever, and all other symptoms much better, including his breaking back. He received no further medication. Was entirely well the day following save only a slight exhaustion, and his mind was quite at ease on the smallpox question.

Mrs. A. B., æt. 43 years. Nausea, fever, chilliness, aching of knees, throat exceedingly sore and dry, neck very sensitive to touch, aching of nape, lassitude and exhaustion. Inspection of throat showed inflammation, with several points of ulceration, tonsils enlarged, left one most so. *Lachesis* C.M. (Fincke) was given, one dose, followed by the usual *Placebo.* The *next* day a slight fever, and very little soreness of the throat, was all that was left of her troubles. She was well, and about her household affairs two days after.

W. H., aged twenty-seven years, gave the following record on the 9th of August, '80. Has had dysentery for eight years. Stools contain blood and mucus, from six to twelve daily, much tenesmus, burning in anus after stool, sudden urging to stool on awakening in morning; he is of a stooping figure, and greatly emaciated. Prescribed one dose of *sulphur* M.M. (Bœricke). Aug. 15 reported that a decided improvement had taken place in a day or two, and continued for two more days; he is not losing ground, but not improving. Gave one more dose of *sulp.* M.M. Aug. 28. Feels much better, and improvement continues. *Sac. Lac.* Sept. 11. Improvement, which ceased five days ago. One dose *sulp.* M.M. Sept. 24. Has felt "entirely well" until within three days, when having taken a severe cold, the trouble slightly returned. The symptoms of his "cold" not contradicting *sulp.* I gave him one more dose of the same (M.M.) which was all the medicine he has needed. January 30, '81. Applied for treatment for another cold, much severer than the last, which has affected his throat principally but not at *all* the old trouble. His bowels now move regularly once a day, without pain, mucus or blood, and from the patient's general improved appearance one could scarcely doubt his word when he declared that he "never felt better in his life *down there.*" This patient had no change of diet, as that had been already suggested to him by his many allopathic and pseudo-homœopathic physicians, and tried without avail.

J. M., æt., 32. Had been treated by several physicians, who had pronounced his case one of incipient phthisis and *incurable.* Great dyspnœa, especially left side; left lung obstructed, night sweats; a sharp pain in region of liver on deep breathing. Great tremor of body; nervousness; voice so weak can scarcely talk; hard cough; pulse 108; a dull pain in lower left lung, and great sensation of pressure there. Sleeps fairly till 4 A.M. then awakens with cough and can not sleep after. Prescribed *kali carb.* 4 M. (Fincke) a dose every two hours till bed time, (probably about *four* doses). Twelve days after called and stated that he felt "wonderfully better." Still has some cough but less than here-to-fore. Yellowish expectoration; no dyspnœa; appetite good, sleeps well all night till six o'clock, and no perspiration. Pain in right side gone, also the tremor and weakness of voice, pulse 96. *Sac. Lac;* called, fifty-five days after, stating that he had sweats slightly for two nights, appetite not quite so good. Bowels costive, some expectoration and much lassitude. Does not cough often but hard. One dose of *phosph.* 19 M. (Fincke) was all the medicine given, and he has had none since, (two months).

I have thus given a few cases which presented no signs of self-cure; which were, in fact, getting worse daily. Several of the number very acute in nature, and threatening violence. One of them markedly chronic and having for a number of years resisted many other forms of treatment. And all "*by chance*" disappearing under *fluxion* potencies and moderately high ones at that. I can see but one complaint that my friend can enter against these reports, and that is that they were not sufficiently lucrative. I would suggest, however, that this need be no bar to his attempting a little such "*shamming*" as he could make more *frequent* prescriptions of "*sugar* pills." If these medicines *be* a "*sham*," then also is homœopathy a sham, life, light, health and food, and our very beings. I have faithfully "tried and found wanting," low potencies and frequent medication, and I "know whereof I speak" when I say that I am willing to stake reputation, the lives of my patients, and my own life, if need be, on that which has conquered where nought else could.

